# A Visit—Educational

**Published:** 1872-02

**Authors:** 


### A VISIT—EDUCATIONAL.

We recently enjoyed the privilege of a flying visit to several of our Dental Colleges, and saw many things that were gratifying indeed. To see so many young men in active training fo the practice of our profession was pleasant. In Philadelphia, the number of students is nearly equally divided between the two schools, each one having a larger number than any other Dental College in the country. Whether this is a consequence of the greatly increased facilities there for attaining thorough instruction, we shall not attempt now to decide. It has, however, more than once been said in our hearing, that their teachers are men of large hearts and generous impulses. Much has been done in both these institutions in the way of procuring accessory apparatus and appliances for illustration. In reference to the clinical department, as there presented, the question forced itself upon us, would it not be preferable to have less in quantity, and a better quality?

The New York College of Dentistry was in active operation, with a good class. All its departments seemed to be moving along energetically. This institution, however, labors under the difficulty of having very little sympathy from the profession in the city of New York. Whether this is to be attributed to the profession there, or to something in the college, we are unable to determine. But it is beyond all question that, could the co-operation, sympathy and support of the dental profession of New York and its vicinity be concentrated to the support of a Dental College, such a result would be attained as has never existed in this direction, and will never be witnessed elsewhere for a long time to come. The wealth and men of ability for such an enterprise are there, and every one has a desire for the development, growth and progress of the profession; but from whence shall emanate the power to harmonize the elements?

The New York College of Dentistry is doing a good work for a few, while it ought to do a better work for hundreds.

In Boston there are two Dental Colleges, viz.: the Boston Dental College and the Harvard Dental College. These schools are both doing a good work, so far as they have the means. The Harvard College, however, has much the advantage of its compeer in this respect. In its facilities for medical teaching, it has all that Harvard can give. Its corps of dental teachers are excellent men, who will doubtless avail themselves of everything that will aid in giving most thorough instruction to their pupils.

This institution will ultimately, without doubt, be very efficient indeed, if it is not dominated or kept too much in the background by the other interests of the University; there need not, however, be occasion for anything of this kind.

The Boston Dental College is accomplishing a good work, though under great disadvantages. It has been but recently organized, and has no sustaining influence or power behind it, except in the energy and special effort of those immediately interested, and they are realizing what it is to build up an institution *de novo*, by individual effort.

Each and every one of our Dental Colleges is doing a great work for the profession, and they should receive far greater encouragement and support than have as yet been experienced by any, so far as we are aware. Every member of the profession should take up the interest of some one of our colleges, and give it true, hearty and material support in every way possible. The whole profession becoming thus interested, our institutions would far more nearly fulfill their mission—effecting much more good than hitherto.

One great difficulty under which all our colleges labor is, that they are regarded by the profession too much in the light of individual enterprises. No institution of this kind, that is dependent for its prestige and development upon one, two, or a very few individuals, can be upon a very firm and enduring basis; but when the cause, in the interest of which they are established, is of such magnitude and importance that the individuality of men, however great and strong they may be, is merged into and seem only as a constituent part of the institution, then may the hope be entertained that it will not pass away when the man dies, but live through successive generations, and its influence go forth, not for the promotion and aggrandizement of the few favored ones, but for the welfare and best interests of all who may come within the sphere of its operations.

In view of these things, then, let every member of the profession rally definitely, and with will and purpose, to the support of some one of our colleges; and let it be no half-way support, but an earnest, efficient laboring in behalf of the cause you espouse; and not only this, but in view of your effort, demand that the work shall be better done than ever before—that progress shall be made, and that the standard shall be borne higher and higher each succeeding year, until there is a much larger measure of ability in those who assume the responsibilities of our profession, to rescue and deliver these broken and tortured bodies of ours from the thralldom of disease and death.

